# AI-powered classification and network analysis for knowledge mapping in medicine: a century of neurosyphilis research

**DOI:** 10.1186/s12874-025-02750-8

**Published:** 2025-12-26

**Authors:** Justine Falciola, Myriam Lamrayah, François R. Herrmann, Alexandre Wenger, Laurence Toutous Trellu

**Affiliations:** 1 Department of Rehabilitation and Geriatrics, Faculty of Medicine, Geneva University Hospitals and University of Geneva, Geneva, Switzerland; 2 Faculty of Medicine, Geneva University Hospitals and University of Geneva, Geneva, Switzerland; 3 Institute for Ethics, History and the Humanities, University of Geneva, Geneva, Switzerland; 4 Department of Dermatology and Venereology, Faculty of Medicine, Geneva University Hospitals and University of Geneva, Geneva, Switzerland

**Keywords:** Neurosyphilis, Syphilis, Data network, Large language model, Knowledge mapping, Scoping review

## Abstract

**Background:**

Tracking the evolution of scientific knowledge is challenging due to the scale and complexity of the biomedical literature. Neurosyphilis is a clinically complex and historically stigmatized condition that remains difficult to diagnose and manage. Its underexplored literature offers an ideal test case to evaluate digital methods for mapping research trends and identifying knowledge gaps. We aim to assess how large language models (LLMs), network analysis, and interrupted time series analysis (ITSA) can be combined to automate literature classification and examine how knowledge of neurosyphilis has evolved.

**Methods:**

We systematically searched Web of Science, Embase, PubMed Central, the Cochrane Library, and Lens for records on neurosyphilis published until December 31, 2024. We included records with available titles and abstracts in which GPT-4o mini was identified as being focused primarily on syphilis or neurosyphilis. Eligible records were classified into 23 research fields via LLM-based prompts. Network analysis visualized changes in research structures over time, and the ITSA assessed associations between publication trends and major clinical or technological milestones.

**Results:**

Among the 14 934 retrieved records, 4 646 met the inclusion criteria. LLM-based classification showed high repeatability (agreement = 99·67%, 95% CI 99·47–99·80; Cohen’s κ = 0·99, 95% CI 0·96–1·00). Biomedical, Clinical, and Health sciences were the most common domains. Network analysis revealed a shift from dense, discipline-specific clusters to larger interdisciplinary structures. ITSA revealed significant increases in publication activity following the introduction of penicillin G, HIV emergence, genome sequencing of *Treponema pallidum*, and the rise of digital dissemination platforms.

**Conclusions:**

Combining LLMs with bibliometric and network methods provides a scalable framework for analyzing large-scale biomedical literature. When applied to neurosyphilis, the approach revealed links between research activity and clinical and technological advances. In addition to this case study, the method could support meta-research and inform evidence-based decision-making across other complex medical conditions.

**Supplementary Information:**

The online version contains supplementary material available at 10.1186/s12874-025-02750-8.

## Background

Systematic literature reviews are essential for biomedical research, but the sheer volume and pace of new publications increasingly overwhelm traditional review workflows. Large language models (LLMs), AI systems trained on massive text corpora, have shown human-comparable performance in natural language understanding and classification tasks [1]. Their use in healthcare management is expanding rapidly [2], yet their ability to support large-scale bibliometric analysis for a disease-specific domain remains underexplored.

In this study, we present and evaluate a scalable, AI-assisted bibliometric framework powered by LLMs to classify, structure, and interpret biomedical literature. We apply it to neurosyphilis (NS), a well-suited case study due to its manageable publication volume, historical depth, and multidisciplinary relevance arising from its complex biological and clinical features. While known since the 15th century, syphilis remains a major public health concern and a priority for the World Health Organization, with eight million adults contracting it in 2022 [3]. Neurological complications arise from bacterial invasion of the central nervous system, a stage that remains incompletely understood owing to diagnostic, biological, and historical complexities [4]. From a diagnostic perspective, this severe form of syphilis infection needs rapid and reliable detection, which is particularly challenging in asymptomatic or latent stages. Then, cerebrospinal fluid analysis can yield inconclusive results in the absence of a gold standard biomarker to confirm the medical hypothesis [5]. Furthermore, the lumbar puncture to collect cerebrospinal fluid is a complex procedure requiring careful training. Biologically, the pathophysiological mechanisms of infection remain unclear because of the difficulties to cultivate in vitro the pathogen *Treponema pallidum* [6]. Since its complete genome sequencing in 1998 [7], genomics studies progressively highlight the strain diversity that may affect virulence and host interactions which encourages the scientific community to stay attentive in clinical practice and pursue fundamental investigations [8]. Historically, NS research has been hindered by social stigma, particularly due to its association with psychiatric symptoms, its main mode of sexual transmission, and the fragile affected populations (sex workers, men who have sex with men, drug users, incarcerated). Despite modern technological advancements improving investigations for neurodegenerative diseases such as neuroimaging (MRI, PET), genomics (next-generation sequencing), in vitro models (brain organoids), microscopy (tissue clearing), critical aspects of neuro-infections and NS management remain unresolved.

Given these limitations in available diagnostic tools and therapeutic options, a comprehensive analysis of the NS literature is essential to track scientific progress and identify areas requiring further research. We adopt the premise, established in the fields of bibliometrics and science mapping, that patterns in scientific publications can serve as a proxy for the evolution of medical knowledge. Changes in publication volume, domain focus, and disciplinary overlap provide observable signals about epistemic shifts in the field [9]. The objectives of this study are twofold: first, to develop a scalable, AI-assisted framework for automated literature classification via general-purpose LLM (GPT-4-o mini); second, to analyze the evolution of scientific research on NS by applying this framework. Although both LLM-based text classification and NS have been studied independently, their integration for large-scale bibliometric and network analysis remains unexplored. This study therefore introduces an integrated, reproducible approach that combines LLM-based classification, network analysis, and interrupted time series analysis (ITSA) to map how knowledge structures and research activity have evolved in response to major clinical and technological milestones. While interdependent, these aims represent distinct analytical layers: the methodological validation of the tool and its application to a clinically and historically complex condition.

## Methods

### Data sources

Records published until December 31, 2024, were retrieved between September 19, 2024, and April 4, 2025, from five databases: Web of Science, Embase, PubMed Central (PMC), the Cochrane Library, and Lens. The search strategy was standardized across databases using the subject heading “neurosyphilis” (MeSH or equivalent; Table S1). Records were included if both the title and abstract were available. We define a record as the bibliographic metadata (i.e., title and abstract) of a record indexed in a database, following PRISMA 2020 definitions [10]. Although each record may correspond to a full-text report, only records were used for classification, as full-text access was not consistently available across sources. This approach maximized inclusion while maintaining a consistent input structure for the model. Metadata were exported directly to Excel software (Microsoft Corporation) when supported or extracted via Python software version 3.12 (Python software Foundation) [11]. Duplicates and ineligible records were removed before classification. Deduplication was performed manually with programmatic support using Stata software version 18 (StataCorp 2023. College Station, TX: StataCorp LLC) and RStudio v2025.9.1.401 (Posit Software, PBC, Boston, MA). To enable matching, we generated unique textual identifiers by concatenating and cleaning title and abstract strings (converted to lowercase and stripped of special characters).

### Classification tasks

Two distinct classification tasks were conducted using GPT-4o mini via the OpenAI API (Application Programming Interface, OpenAI, 2025) [12]. The API parameters were set to a temperature of 0·2, a top-p of 1·0, and a maximum token limit of 100. Each call included only a system message (“You are a concise assistant.”) and a user prompt, ensuring stateless, independent interactions. The prompt language and model outputs were in English. No system memory or contextual chaining was enabled between prompts, preserving the independence of each classification instance and satisfying the assumption of independent and identically distributed observations required for interrater reliability statistics.

Tasks were parallelized across two computers via separate API keys to manage computational demands. The dataset was divided into multiple Excel input files and processed in batches over two weeks. Because each instance was evaluated independently with a clean prompt history, this setup did not compromise reproducibility or the statistical independence of observations. All outputs were saved alongside prompt metadata in structured datasets.

- Task 1: Topical relevance screening. Each record, consisting of the title and abstract only, was screened to determine whether syphilis or NS was a central theme. The prompt was implemented in Python 3.12 (Windows 64-bit) with OpenAI 0.28.0, Pandas 2.2.2, and NumPy 1.26.4. Sampling used a fixed random state (42).
- Task 2: Research field classification. Each record retained after Task 1 was classified into one or more of the 23 research fields defined by the Australian and New Zealand Standard Research Classification (ANZSRC) [13]. The task was executed in Python 3.12 (Windows 64-bit) with OpenAI 0.28.0, Pandas 2.2.2, and standard libraries (Time, Pathlib). Each record was processed deterministically using identical prompts and logged API parameters, enabling full reproducibility of GPT outputs. Each prompt (23 in total, one per field) included the name, formal definition, and exclusion criteria of the research field, followed by the title and abstract of the record.

To assess classification repeatability, both tasks were conducted twice at separate time points via the same prompt templates and parameters. Agreement between repetitions was calculated (see section Statistical analysis). Instances of disagreement were manually and independently reviewed by the authors (JF and ML, blinded to GPT justifications) and resolved by consensus. All the scripts were run on Python using PyCharm version 2023.3.2 [14]. The full prompt logic and model configurations used in the workflow are available in the project repository (https://github.com/JUFALC/Neurosyphilis-network/tree/main) and upon request to support reproducibility. 

### Method assessment

Model selection and validation were conducted to evaluate the reliability of using GPT-4o mini for the automated classification approach. To empirically assess this choice, independent re-classifications were performed on a randomly selected 10% validation subset of the full dataset (n= 541 records). This subset was reprocessed using GPT-4o mini, GPT-4o, GPT-5 mini, and manual human coding to benchmark agreement, runtime, cost, and the trade-off between sensitivity and specificity. All AI-classifications were conducted with same parameters (OpenAI API; temperature = 0.2, top-p = 1.0).

Validation included two complementary tasks. Task 1 evaluated topical relevance, comparing model and human classifications for whether each record primarily addressed syphilis or NS (n = 541). Task 2 assessed multi-label fields of research classification for the subset of 471 records identified as syphilis- or NS-focused in Task 1.

Comparative performance metrics are summarized in Table S2 for Task 1 and in Table S3 for Task 2. In both cases, agreement with the baseline GPT-4o mini model, runtime, and estimated processing costs were recorded to evaluate efficiency and reproducibility of our retained model. Model costs were estimated using OpenAI’s October 2025 pricing (GPT-4o mini: $0.00015 / 1k input tokens, $0.0006 / 1k output; GPT-4o: $0.005 / 1k input, $0.015 / 1k output; GPT-5 mini: $0.00025 / 1k input, $0.002 / 1k output). For manual classification, we considered the Swiss salary of a master’s-level research assistant (CHF 35/hour ≈ $37 USD/hour), which aligns with standard rates across Swiss universities.

For Task 2, a subset of four fields of research (Biological, Biomedical/Clinical, Chemical, and Health Sciences) was extracted from the total of 23 to analyze in greater detail. These categories are not statistically representative of all 23 fields of research, but they represent the most frequently assigned and interconnected domains within the corpus, consistent with the predominantly biomedical focus of the literature on syphilis and NS. Focusing on these major categories provides clearer insight into classifier behavior in the most information-dense areas of the dataset.

Fig. S1 presents the ROC curves for Task 1, showing correspondence between LLM-generated classifications and human reference coding.

Fig. S2 illustrates model performance for Task 2 across the four main fields of research: (a) Biological, (b) Biomedical/Clinical, (c) Chemical, and (d) Health Sciences. Each panel reports sensitivity, specificity, and ROC area (AUC) for baseline GPT-4o mini, GPT-4o, and GPT-5 mini compared with the human reference.

### Co-occurrence network construction

Each record was represented as a set of assigned research fields. For every pair of fields co-occurring within a single record, an undirected edge was generated. Self-loops were added for single-field records to preserve information on isolated domains. The resulting dataset of node pairs was annotated with the year for longitudinal analysis.

### Network metrics

All network construction and visualization were performed in Python 3.12 (Windows 64-bit) using Pandas 2.2.2, NumPy 1.26.4, NetworkX 3.3, Matplotlib 3.9.2, Seaborn 0.13.2, Plotly 5.24.1, and OpenPyXL 3.1.5 [15, 16]. Random layouts were generated with a fixed seed (42) to ensure identical graph structures and figures across runs. In each graph, nodes represent research fields, node sizes reflect record frequency, edges denote co-occurrences, and edge widths indicate co-occurrence strength, with visual scales adjusted annually. For temporal comparison, the study period (1916–2024) was divided into six historically informed epochs reflecting major milestones in scientific communication, from early discipline-bound research to the recent integration of AI tools. This predefined framework guided subsequent network analyses.

### Interrupted time series analysis

To evaluate the impact of major historical advances on annual publication trends, we conducted interrupted time series analysis (ITSA). The selected milestones represent well-documented turning points in clinical management and scientific communication that are likely to have influenced neurosyphilis research. Clinical milestones included the introduction of penicillin G (1945–46), the emergence of HIV (1981), and the genome sequencing of *Treponema pallidum* (1998). Technological milestones included the launch of MEDLINE (1971), PubMed Central (1999), and the rise of open-science repositories after 2010.

Models were estimated in Stata via the itsa command [17, 18], and changes in level and trend were assessed for each time point.

### Statistical analysis

For each classification task, two independent experiments were run. Agreement between experiments was measured via raw concordance and Cohen’s kappa coefficient (κ) with corresponding 95% confidence intervals (CI) [19]. The κ values were interpreted according to conventional thresholds: < 0·20 (slight agreement), 0·21–0·40 (fair), 0·41–0·60 (moderate), 0·61–0·80 (substantial), and > 0·80 (almost perfect agreement) [20]. Statistical significance was tested using the two-sided z test for H₀ : κ = 0 performed by Stata’s kap command.

Structural network evolution over time was assessed by computing descriptive network statistics via Python’s library. This included degree centrality (number of direct connections), Katz centrality (influence on the basis of both direct and indirect links), the clustering coefficient (the tendency of nodes to form tightly connected groups), and betweenness centrality (the extent to which a node serves as a bridge between others). Global network metrics, including the number of nodes and edges (network size), network density (the proportion of observed connections relative to possible connections), average (avg) path length (the typical node-to-node distance), and network diameter (the longest of all shortest paths between any two nodes in the network, reflecting its maximum separation), were also computed.

ITSA was conducted using Poisson regression model with a log link function [21], and Newey-West standard errors with a lag of 1 to account for autocorrelation.

## Results

### Constitution of the corpus

**Fig. 1:**
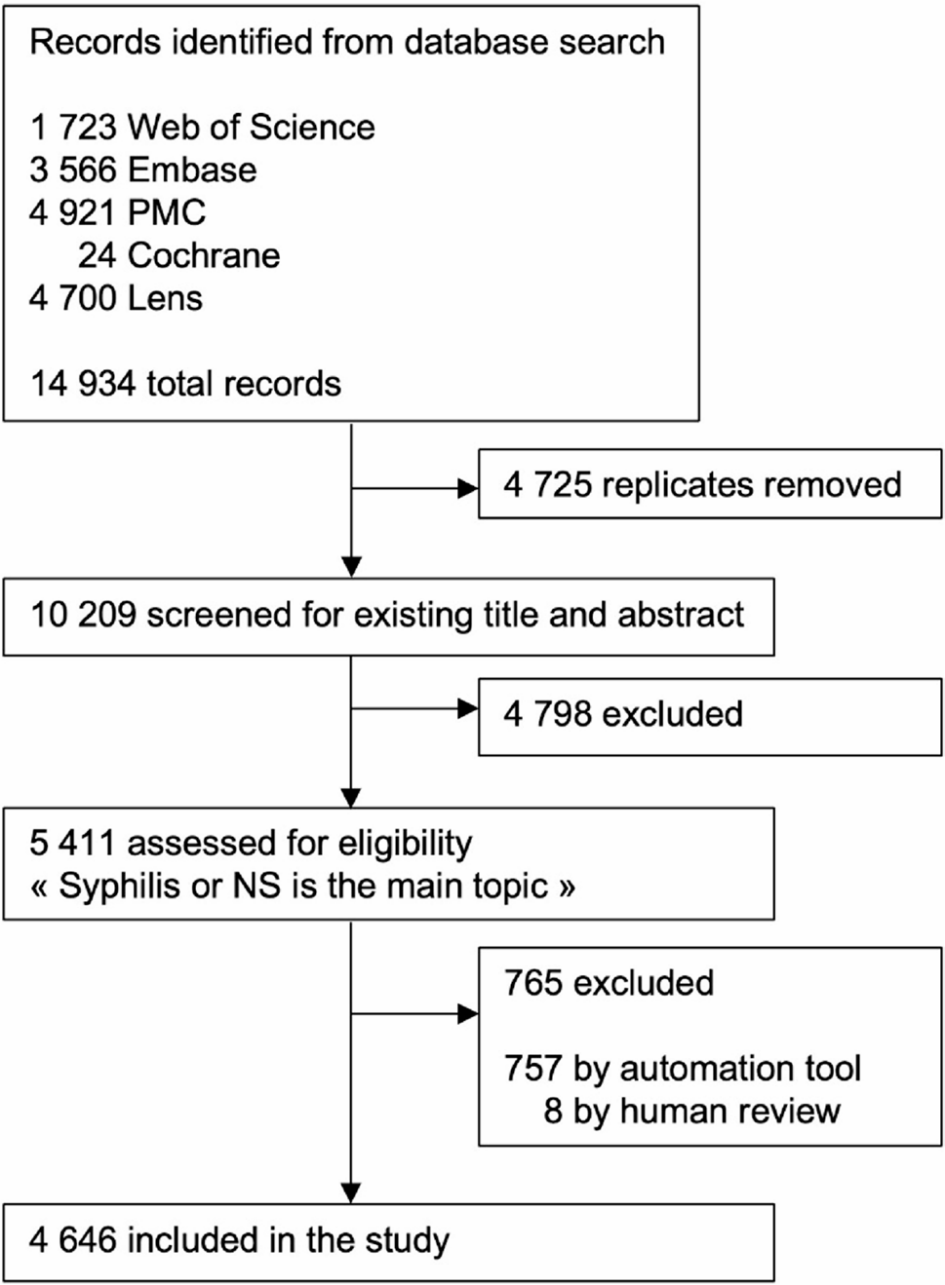
A diagram illustrating the steps employed for the study selection. The flowchart follows the PRISMA 2020 guidelines for systematic reviews

Searches via the MeSH “NS” identified 14 934 records across five databases: Web of Science, Embase, PMC, the Cochrane Library, and Lens. The extracted data were merged into a single dataset, and six variables were retained for descriptive analysis: authors, title, abstract, year of publication, type of publication, and language. After removing replicates and ineligible records, 5 411 unique records, consisting of the title and abstract only, were screened for topical relevance via the GPT-4o mini model. Each record was screened twice to assess classification consistency. The overall agreement was 99·67% (95% CI 99·47–99·80), with an expected agreement of 75·70%. The Cohen’s κ of 0·99 (95% CI 0·96–1·00) indicates “almost perfect” agreement, as per standard interpretation (Table S4). In 765 records, syphilis or NS was not the main topic, and these records were excluded. When needed, instances of disagreement (eight records) were manually reviewed and resolved through consensus. The final corpus included 4 646 records (Fig. 1).

To assess the reliability and efficiency of the classification framework, comparative validation was performed across models and reviewers, as summarized below.

### Model performance

The OpenAI family of models was selected for its stability and widespread use in academic research. Given the binary nature of the classification tasks, GPT-4o mini, chosen for its balance of accuracy, cost-efficiency, and reproducibility, was expected to perform comparably to larger models while remaining faster and more economical to scale.

Across both validation tasks, GPT-4o mini achieved strong agreement with human coding while remaining substantially faster and less costly than larger models. As shown in Fig. S2, it demonstrated the most balanced and stable performance, with high sensitivity (≥ 0.98) and moderate-to-strong specificity (up to 0.98). All models tended to favor sensitivity over specificity, consistent with the design goal of maximizing recall and minimizing the risk of missing relevant records.

These findings should be regarded as proof of concept rather than definitive validation. With further prompt optimization or domain-specific fine-tuning, other models could achieve different or improved trade-offs. In its present configuration, however, GPT-4o mini provides a practical, transparent, and scalable baseline for large-scale, inclusive literature screening.

### Characteristics of the corpus

**Fig. 2:**
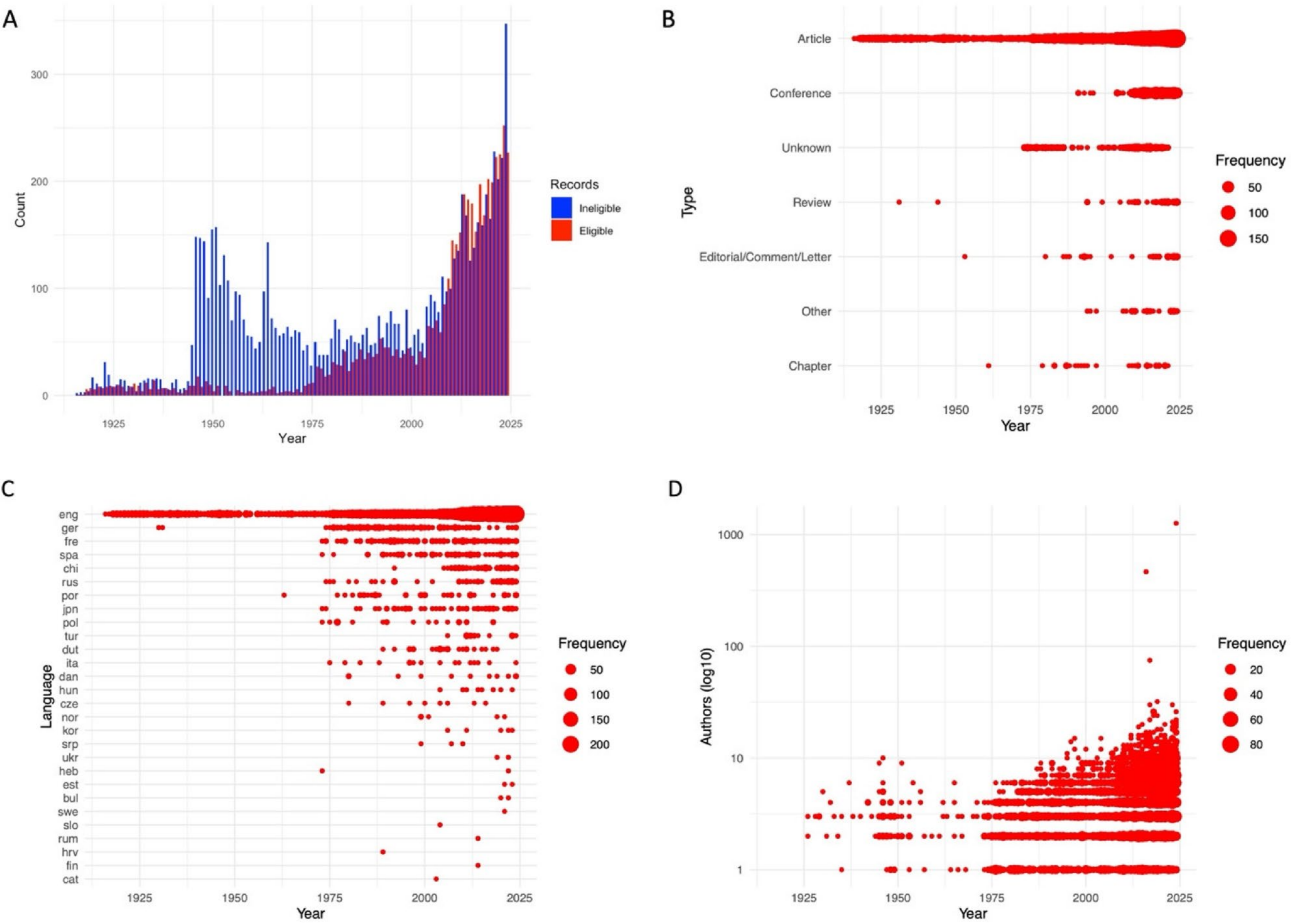
Descriptive analysis of the corpus. **A** shows the global distributions of the included (red) and excluded (blue) corpora over time; a focus on the distribution of included records is made by publication type (**B**), by language (**C**), and by coauthor number (**D**). Bubble sizes represent the relative frequency of occurrence for each category, highlighting trends in record attributes

The 4 646 included records were published between 1916 and 2024, with two notable peaks in frequency: the first peak occurred in 1976 (27 records), and the second peak occurred in 2009, when the annual output exceeded 100 records for the first time. Article papers were the most common type (77·9%), followed by conference proceedings (14·8%). English was the predominant language (86·1%), with smaller contributions from German (2·7%) and French (2·5%). Other languages collectively account for less than 9% of the corpus. The number of coauthors progressively increased with time, reaching more than seven contributors on average for 2024. A detailed breakdown of the included corpus characteristics is provided in Fig. 2.

To evaluate potential selection bias resulting from the inclusion of only records with online titles and abstracts, we examined the 4 798 ineligible records. These records spanned the years 1916–2024, with 58% published prior to 1975. Most records were classified as article papers (88·9%). The language field was frequently unspecified (labeled “und”), primarily due to the absence of abstracts. Corresponding descriptive metrics are available in Fig. S3.

### LLM research field classification performance

The 4 646 included records were classified across the 23 ANZSRC research fields via GPT-4o mini. Each record was classified twice at separate time points to assess consistency. The agreement across rounds ranged from 95·42 to 100·00% (Table S4). High Cohen’s κ values (≥ 0.8) across all fields indicate “almost perfect” agreement. All comparisons were statistically significant (*p* < 0·001), rejecting the null hypothesis that agreement occurred by chance.

### Distribution of research fields

**Fig. 3:**
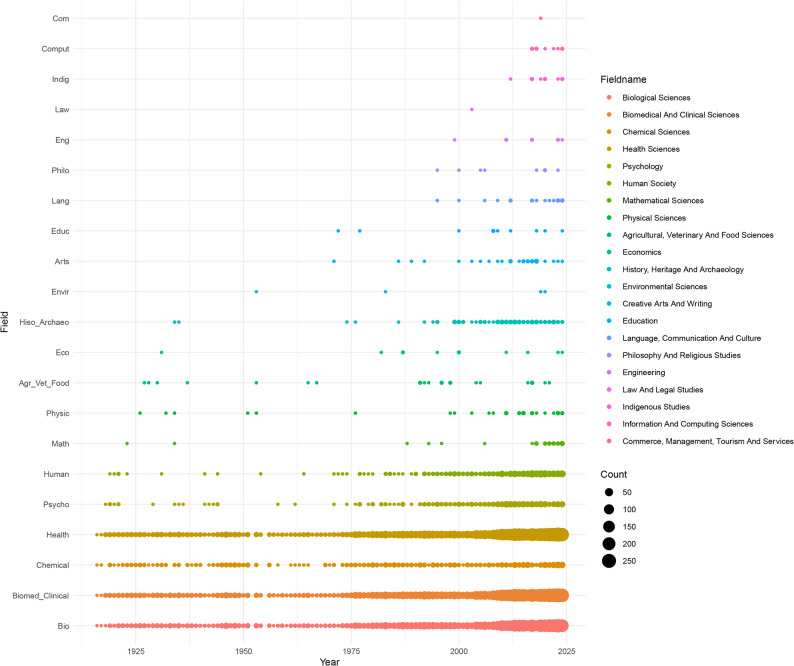
Evolution of the number of records per research field from 1916 to 2024. The 21 cited research fields are presented in chronological order based on the first year of occurrence

The three most frequently assigned research fields were “Biomedical and clinical sciences” (4 608 records), “Health sciences” (4 607) and “Biological sciences” (3 714). These were followed by “Chemical sciences” (470), “Human society” (458) and “Psychology” (393). Two research fields (“Build environment and design” and “Earth sciences”) were never assigned. All remaining research fields were found in fewer than 40 records. Among the 21 research fields, nine began to appear after 1970. Overall, NS research remains concentrated in biological domains, with limited engagement across certain disciplines (Fig. 3).

### Network analysis

We constructed longitudinal co-occurrence networks to explore how disciplinary links evolved in NS-related research. The resulting structures reveal a transition from small, dense, and locally clustered networks to larger, more distributed, and interdisciplinary systems. A dynamic visualization of the network’s evolution over time is available in the GitHub’s project repository.

**Fig. 4:**
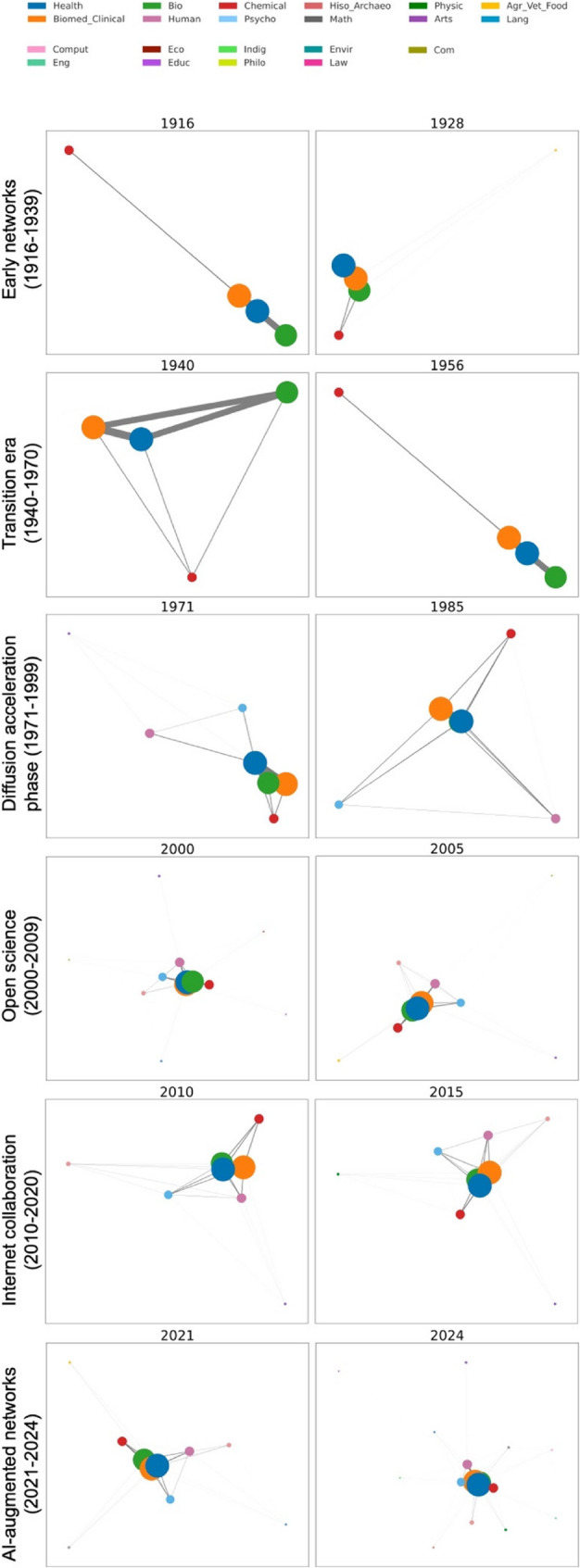
Evolution of research networks from 1916 to 2024. Each row corresponds to one of six historically informed epochs—ranging from early discipline-bound networks (1916–1939) to the recent AI-augmented period (2021–2024)—defined according to major milestones in scientific communication and collaboration, as well as structural inflection points observed in the co-occurrence networks. Two representative years within each epoch are shown to illustrate structural changes in network size, density, and connectivity

To facilitate interpretation, the research period (1916–2024) was divided into six epochs corresponding to key time points in the evolution of knowledge diffusion (Fig. 4; Table 1).

**Table 1:** Descriptive statistics of networks for each time period

	Rec (min; max)	Nodes	Edges	Diam	Dens	Length	Degr	Centr	Clust	Btw
Early networks (1916–1939)	7·08 (1; 14)	4·92	8·83	1·50	0·91	1·09	3·47	0·91	0·94	0·03
Transition era (1940–1970)	5·76 (2; 18)	4·28	6·79	1·28	0·95	1·05	3·06	0·95	0·96	0·01
Diffusion accel. era (1971–1999)	30·35 (3; 53)	6·59	14·31	1·90	0·77	1·23	4·20	0·77	0·88	0·05
Open science era (2000–2009)	59·10 (29; 108)	9·10	23·30	2·40	0·64	1·39	5·05	0·64	0·83	0·05
Internet collab. era (2010–2020)	173·27 (141; 202)	10·91	34·64	2·09	0·67	1·34	6·25	0·67	0·86	0·04
AI-augmented era (2021–2024)	231·75 (223; 252)	13·50	44·50	2·25	0·54	1·48	6·53	0·54	0·82	0·04

This table summarizes the average network statistics across six time periods, reflecting key historical and research advancements. Metrics include the average number of records (rec), nodes, edges, and structural characteristics such as network diameter (diam), density (dens), average path length, degree (degr), centrality (centr), the clustering coefficient (clust), and betweenness centrality (btw). Each metric is averaged over the years within each period

In the early networks (1916–1939), the average density (0·91) and clustering coefficient (0·94) were high, whereas the diameter (1·50) and average path length (1·09) remained low, reflecting tight internal connectivity within small networks. During the transition era (1940–1970), network size remained modest, with slightly lower density (0·95) and clustering (0·96) and a small decrease in betweenness centrality (0·01), suggesting continued internal cohesion with limited cross-linking.

From 1971 onward, the diffusion acceleration era presented larger networks (average of 30·35 records/year), higher node and edge counts, and increases in diameter (1·90), path length (1·23), and betweenness centrality (0·05). These trends continued into the open science phase (2000–2009), which showed further growth in records (59·10/year), edges (23·30), and diameter (2·40), accompanied by a decline in density (0·64).

In the internet collaboration era (2010–2020), the number of records sharply increased (173·27/year), whereas clustering remained high (0·86), and the density stabilized at 0·67. The AI-augmented network phase (2021–2024) reached the largest network size (231·75 records/year), highest degree (6·53), and longest average path length (1·48), whereas density fell to its lowest point (0·54).

Field-level analysis revealed that cross-disciplinary connections increased significantly for biomedical and clinical sciences, health sciences, and biological sciences. In contrast, chemical sciences remained relatively static across all periods. These patterns illustrate a sustained shift from disciplinary insularity to broader field integration in NS research.

### Trends in research activity (ITSA)

**Fig. 5:**
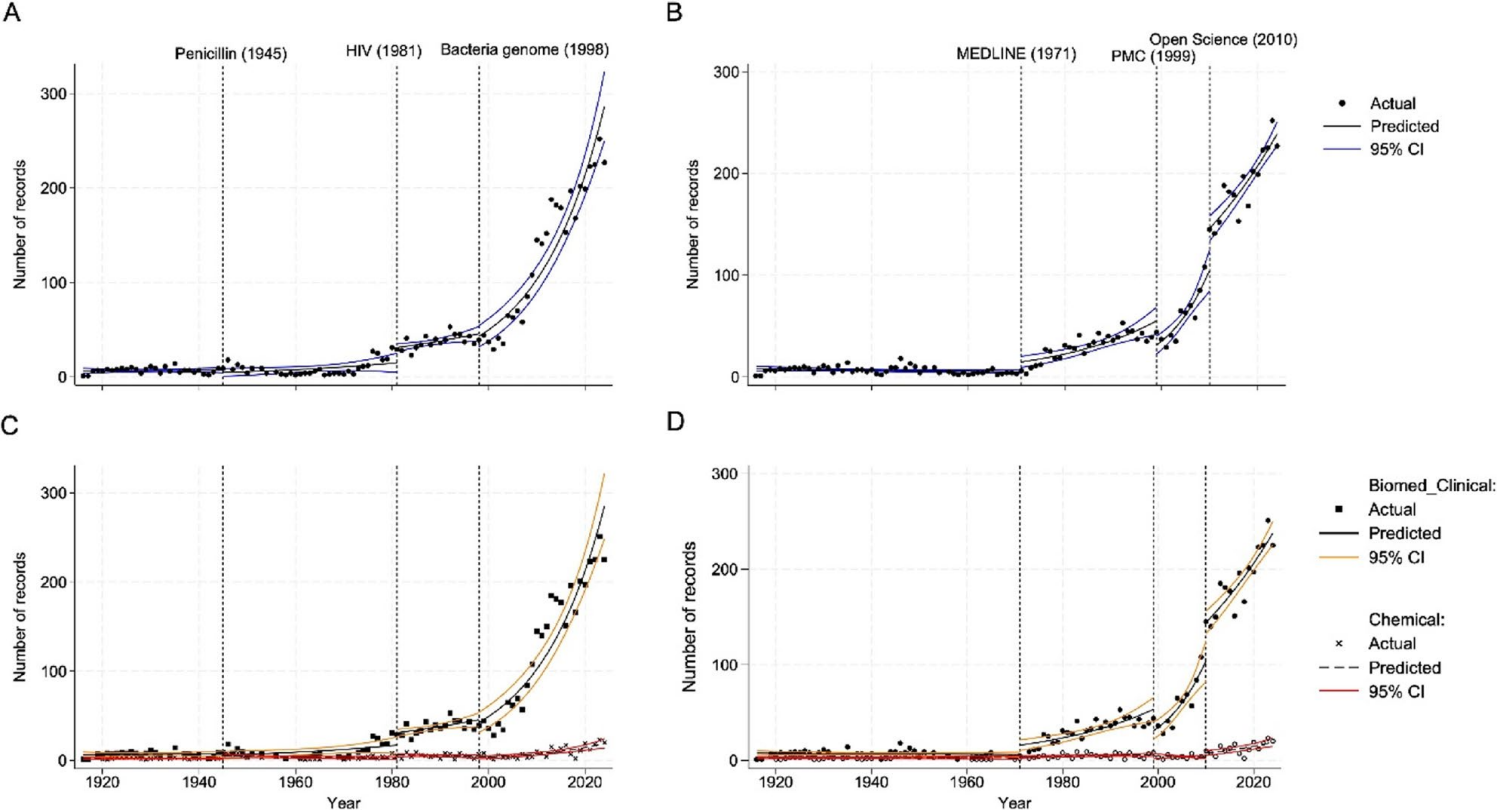
Trends in the number of records highlighting key clinical milestones. Dotted vertical lines mark significant clinical events (**A**, **C**) and technological advancements (**B**, **D**). The graphs display actual record counts (black dots), predicted trends (solid black line), and 95% CI (blue lines), illustrating significant increases in postevent trends (**A**, **B**) and differences in postevent trends between research fields (**C**, **D**)

We used the ITSA to evaluate the impact of major clinical and technological advances on NS research activity over time. The analysis did not reveal statistically significant immediate effects following key clinical milestones (Fig. 5; Table 2, upper panel).

**Table 2:** Statistical analysis of the ITSA

Model	Time Period	Coeff	Coeff value	Std Error	*p* value	95% CI Lower	95% CI Upper
Clinical discoveries							
	Pre 1945	Initial level	6·43	1·400	< 0·001	4·20	9·85
	Pre 1945	Trend	1·00	0·013	0·789	0·98	1·03
*Penicillin G*	1945	Change	0·66	0·322	0·396	0·26	1·72
	Post 1945	Trend	1·03	0·025	0·233	0·98	1·08
*HIV*	1981	Change	2·07	0·737	0·041	1·03	4·16
	Post 1981	Trend	0·99	0·021	0·658	0·95	1·03
*Genome*	1998	Change	0·94	0·148	0·715	0·69	1·28
	Post 1998	Trend	1·05	0·011	< 0·001	1·03	1·07
Postevent linear trends							
	1945		0·03	0·020	0·107	-0·01	0·07
	1981		0·02	0·008	0·003	0·01	0·04
	1998		0·07	0·006	< 0·001	0·06	0·09
Research sharing							
	Pre 1971	Initial level	7·95	1·192	< 0·001	5·93	10·67
	Pre 1971	Trend	0·99	0·005	0·081	0·98	1·00
*MEDLINE*	1971	Change	2·99	0·762	< 0·001	1·82	4·93
	Post 1971	Trend	1·06	0·012	< 0·001	1·03	1·08
*PMC*	1999	Change	0·57	0·115	0·005	0·38	0·84
	Post 1999	Trend	1·07	0·023	0·004	1·02	1·11
*Open Science*	2010	Change	1·38	0·155	0·004	1·11	1·72
	Post 2010	Trend	0·93	0·019	< 0·001	0·89	0·97
Postevent linear trends							
	1971	Postevent trend	0·05	0·010	< 0·001	0·03	0·07
	1999	Postevent trend	0·11	0·020	< 0·001	0·07	0·15
	2010	Postevent trend	0·04	0·004	< 0·001	0·03	0·04

This table presents ITSA results analyzing the impact of key historical events on the number of records. Coefficients (coeff) are displayed in exponential form, representing incidence rate ratios (IRRs). Preevent trends, postevent trends, and changes at intervention points are displayed with their IRRs, standard (std) errors, p values, and 95% CI. Linear parameter testing reflects the postevent trends for each intervention, aiding in interpreting temporal changes in record counts. The number of compared records is *N* = 107

However, long-term postevent trends significantly increased after two of the three breakthroughs. Following the emergence of HIV in 1981, publication trends increased significantly by 2·0% annually (*p* = 0·003, 95% CI 1·0–4·0). The most pronounced effect was observed after the publication of the *Treponema pallidum* genome in 1998 [7], which was associated with a 7·0% annual increase in publications (*p* < 0·001, 95% CI 6·0–9·0) (Table S5). In contrast, while the introduction of penicillin G in 1945 showed a positive trend of 3.0% annually, this change was not statistically significant (*p* = 0·107, 95% CI -1·0–7·0).

With respect to technological shifts in research dissemination, no significant increase in the number of records was observed before the 1970s (Fig. 5; Table 2, lower panel). However, several events were associated with statistically significant changes in both level and trend. The introduction of MEDLINE in 1971 led to an immediate increase in output (IRR = 2·99, *p* < 0·001, 95% CI 1·82–4·93), followed by a sustained annual growth of 5·0% (*p* < 0·001, 95% CI 3·0–7·0). The launch of PMC in 1999 initially led to a significant drop in publication level (IRR = 0·57, *p* = 0·005, 95% CI 0·38–0·84) but was followed by an 11·0% annual increase (*p* = 0·020, 95% CI 7·0–15·0). Finally, the Open Science movement in 2010 was associated with a significant immediate increase (IRR = 1·38, *p* = 0·004, 95% CI 1·11–1·72) and a continued annual growth of 4·0% (*p* < 0·001, 95% CI 3·0–4·0) (Table S5), although at a slower pace than in previous periods.

The impact of clinical discoveries on research activity varies significantly across fields. Following the introduction of penicillin G in 1945, the annual publication growth rate in biomedical and clinical Sciences exceeded that of chemistry by 5·0% (*p* = 0·017, 95% CI 1·0–9·0), indicating a stronger and sustained increase in output. Similarly, although marginally significant, a difference emerged after the HIV epidemic in 1981, with 4·0% faster annual growth in biomedical fields than in chemical fields (*p* = 0·055, 95% CI -0·1–8·0). In contrast, after the 1998 sequencing of the *Treponema pallidum* genome, both fields experienced steep growth, with biomedical at 7·3% and Chemistry at 6·9%, but the difference in postevent trends was minimal (0·4%, *p* = 0·780, 95% CI -0·02–0·03), indicating similar growth rates despite differing absolute levels. Although the biomedical curve visually steepens after 1998, this reflects the continuation of an already strong upward trajectory rather than a statistically distinct acceleration relative to that of chemistry. While postevent trends capture long-term dynamics, both penicillin G and HIV were associated with significant immediate increases in publication levels in biomedical and clinical Sciences (IRR = 2·49, *p* < 0·001, 95% CI 1·51–4·09 for penicillin G; IRR = 2·37, *p* = 0·025, 95% CI 1·11–5·03 for HIV), underscoring their acute field-specific impact.

## Discussion

The newly proposed literature search model leveraging LLMs enables reliable, systematic collection and classification of data, supporting the development of a continuously expanding repository over time. As anticipated, the selected disease test presents particularly noteworthy and relevant characteristics. NS research is characterized by historical rupture, disciplinary fragmentation, and persistent stigma. These forces have influenced not only diagnostic and therapeutic strategies but also how the condition has been studied, funded, and represented in the scientific literature. NS shows the potential of AI-assisted bibliometric analysis to track the evolution of medical knowledge in complex, stigmatized, and interdisciplinary domains. Clinically, NS intersects with neurology, psychiatry, infectious disease, and public health. Socially, it has reflected fluctuating narratives, from moralization to neglect, to renewed interest through the lens of neuroinfectious diseases. These dynamics make NS a compelling case for evaluating how AI-assisted frameworks can detect thematic ambiguity, disciplinary overlap, and long-term research shifts.

Our classification system traced both structural patterns across disciplines and diachronic shifts linked to major turning points (e.g., penicillin G, HIV), revealing long-term trends and neglected areas that may warrant renewed attention. Understanding the trajectory of research —ruptures, revivals, and periods of neglect— is essential for shaping more informed clinical strategies and guiding future investigations.

To our knowledge, this is among the first applications of LLMs for large-scale bibliometric classification in this domain. The scientific literature functions as a proxy for knowledge evolution, as it undergoes peer review, reflects scientific consensus, and captures research advancements [22]. Bibliometric studies show that publication trends closely align with the growth and development of scientific disciplines, making literature analysis an effective tool for tracking the development of fields such as oncology and infectious diseases [23, 24].

We used GPT-4o mini to classify whether syphilis or NS was the central topic of each record and to assign research fields across 23 standardized domains. The resulting classifications enabled a longitudinal co-occurrence network analysis. The early period (1916–1939) was characterized by dense, locally cohesive networks, whereas the subsequent transition era (1940–1970) retained internal clustering but showed limited expansion. The growth observed from 1971 onward coincides with the wider adoption of digital tools and increasing coordination across disciplines. The open science phase resulted in more complex and distributed network structures, whereas the internet- and AI-augmented phases further expanded the network size and interdisciplinary reach. Within this broader structural evolution, field-level dynamics also shifted in distinct ways: biological research became increasingly collaborative, whereas chemical sciences remained more isolated, potentially contributing to therapeutic stagnation.

Longitudinal network analysis has been successfully used in medicine to trace the evolution of HIV research, monitor the emergence of antibiotic resistance, and map the progression of diseases such as congenital syphilis [25]. In this study, we used the ITSA to assess how major clinical and technological developments were associated with changes in research output over time. These associations may reflect shifts in research attention and dissemination capacity, whether specific to NS or part of broader biomedical trends. While some clinical milestones corresponded to significant long-term increases in publication activity, immediate effects were less consistent, likely owing to delays in funding cycles, institutional responses, or shifts in research priorities [26–28]. The emergence of HIV may have also triggered a pathocenosis effect, whereby co-occurring epidemics drew renewed attention to syphilis and contributed to the observed growth in related research [29]. In biomedical and clinical sciences, publication trends generally align with major clinical milestones, reflecting the field’s responsiveness to events such as the introduction of penicillin G, the emergence of HIV, and the sequencing of the *Treponema pallidum* genome. These developments were associated with sustained increases in research output over time, although immediate responses were less consistent, likely reflecting delays in funding allocation, shifts in institutional priorities, or broader structural lags in translating clinical breakthroughs into research agendas. In contrast, chemistry showed minimal variation in both immediate and long-term patterns, suggesting that clinical events had limited influence on research behavior in this field. Divergence between fields was most evident after early clinical milestones, with biomedical research more directly shaped by these advances, whereas chemistry remained relatively stable. In contrast, the 1998 genome publication spurred accelerated growth across both fields, suggesting that some technological breakthroughs may have had broad, cross-disciplinary impacts.

Our study has several limitations. First, GPT-4o mini, selected for its accessibility, has limited capacity for deep contextual memory and biomedical nuance. Although we used structured prompts for conciseness and reported strong interrater agreement (κ > 0·80), prompt sensitivity and token truncation may have affected the outcomes in case of longer-text applications or with different model settings. Moreover, future work could consider model version locking and comparisons with domain-specific alternatives such as BioGPT or Med-PaLM. Second, the exclusion of records lacking abstracts or titles, especially from 1950 to 1970, represents a known gap in the corpus. These omissions could be addressed in future work by incorporating gray literature such as doctoral theses or humanities-focused sources if numerized. However, the stability of record inclusion after 1975 supports the overall representativeness of the dataset. Third, while our framework was applied to NS, its generalizability remains untested. The observed associations between publication patterns and clinical or technological events may reflect broader trends in scientific dissemination, not condition-specific dynamics. However, to contextualize our findings, we reviewed bibliometric trends in HIV research, which has historically paralleled NS in terms of complexity and sociomedical impact. Recent studies revealed similar shifts toward interdisciplinary topics, internationalization, and the growing role of comorbidities and socioeconomic framing [30, 31]. These comparisons suggest that the transformations observed in NS research may reflect broader epistemological trends in the infectious disease literature. Future work should explore diagnostic and therapeutic gaps in NS, especially in light of recurring shortages in reference treatments [32]. LLMs may also be refined to detect emerging scientific trends and inform targeted research strategies.

## Conclusions

This study presents a scalable, AI-assisted framework for tracking the historical evolution of biomedical research. When applied to NS, combining LLMs with network analysis and time series modeling reveals long-term trends in disciplinary focus, highlights delays in research responses to clinical milestones and identifies enduring knowledge gaps with discipline-specific barriers. While the method was applied to a historically complex and underexplored condition, its modular design can support comparative analyses across other disease areas. By bridging computational tools with bibliometric insights, this approach offers an attractive and reproducible strategy for mapping knowledge trajectories and informing future research planning in digital health and beyond.

## Supplementary Information

Supplementary Material 1.

## Data Availability

Data generated or analyzed in this study are included in the published article and its supplementary information files are available at https://github.com/JUFALC/Neurosyphilis-network/tree/main.

## Abbreviations

LLM: Large language model
ITSA: Interrupted time series analysis
NS: Neurosyphilis
AI: Artificial intelligence
PMC: PubMed Central
HIV: Human immunodeficiency virus
