# Supplementary material for: AI-powered classification and network analysis for knowledge mapping in medicine: a century of neurosyphilis research
Source: BMC Med Res Methodol. 2025 Dec 26;26:12. doi: 10.1186/s12874-025-02750-8 (PMC12849313; doi:10.1186/s12874-025-02750-8)
Supplement: Supplementary file 1 — Supplementary Material 1. [file 12874_2025_2750_MOESM1_ESM.docx]

SUPPLEMENTARY APPENDIX

**Fig. S1: ROC curves for Task 1 validation (n = 541). GPT-4o mini, GPT-4o (top panel), and GPT-5 mini (down panel) showed high but preliminary agreement with human-coded references (AUC = 0.91-0.96).**


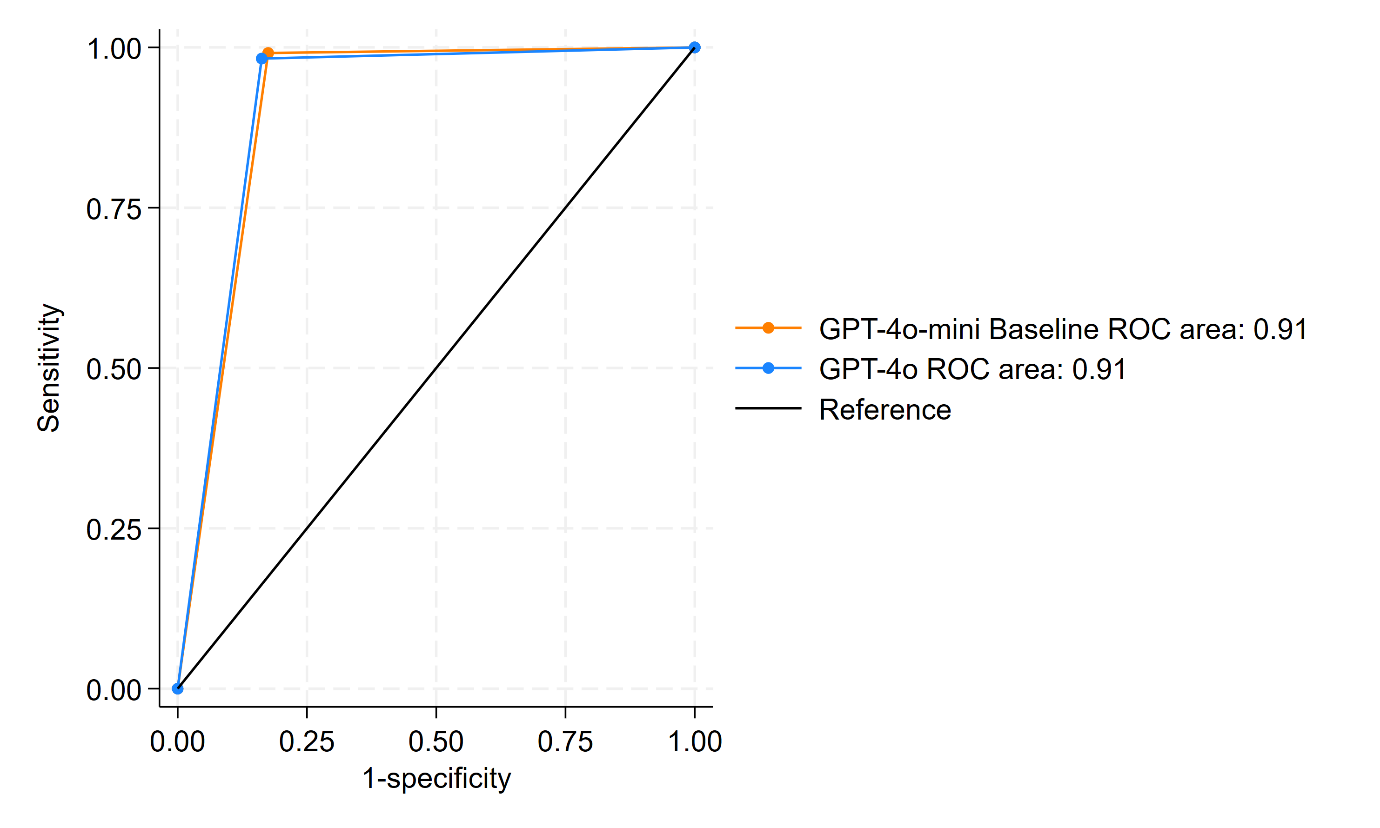


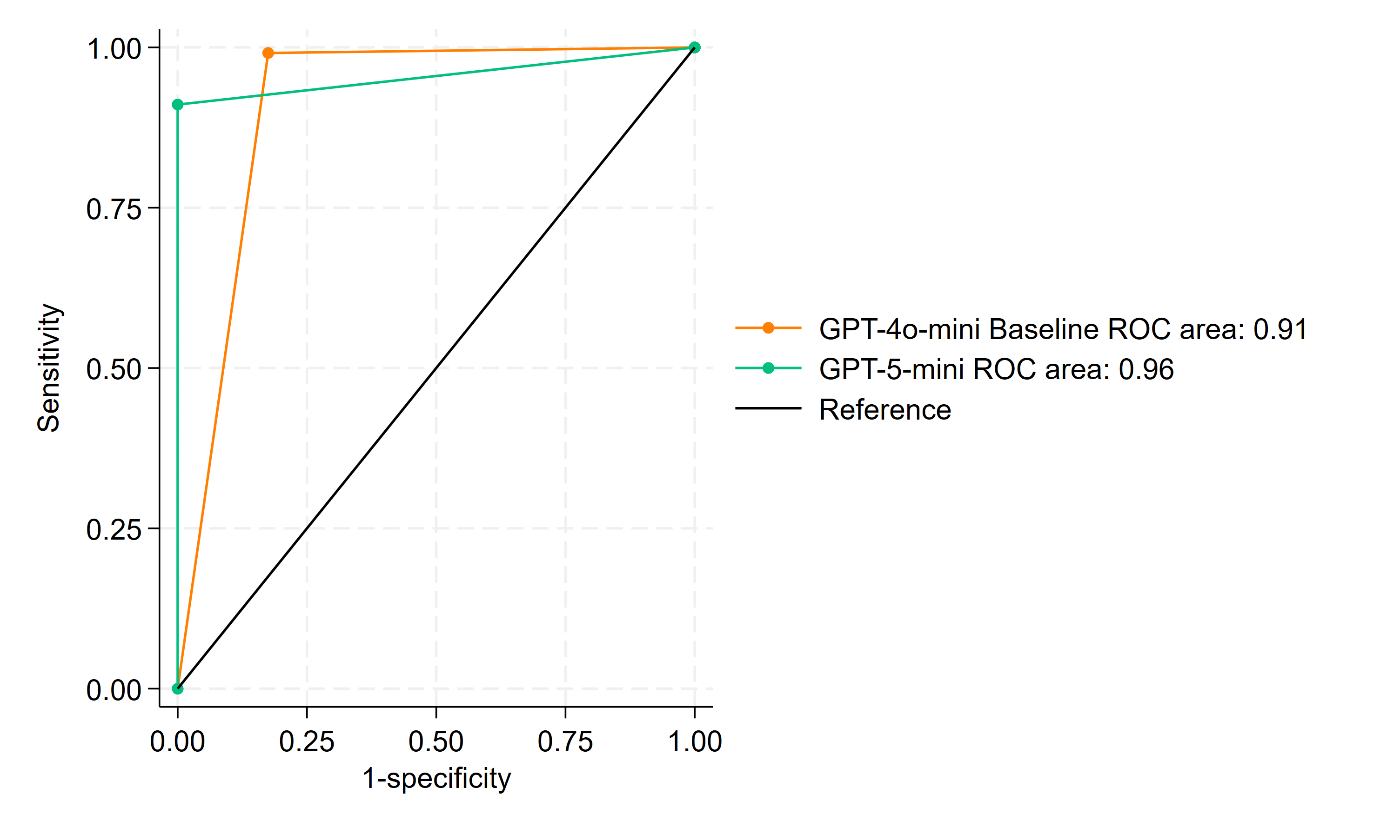


**Fig. S2: Sensitivity, specificity, and ROC area (AUC) calculated for Task 2 validation (n = 471) across the four selected main FoR: (a) Biological, (b) Biomedical/Clinical, (c) Chemical, and (d) Health Sciences.**

1. (b)


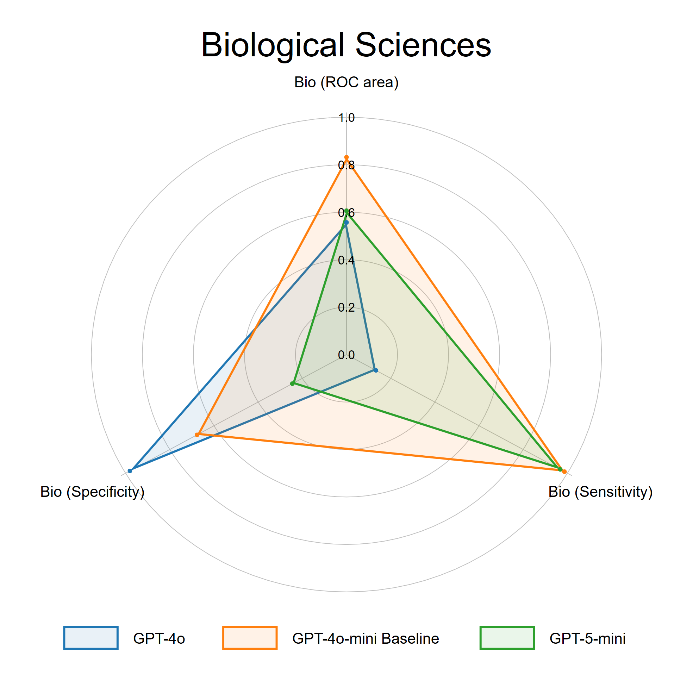

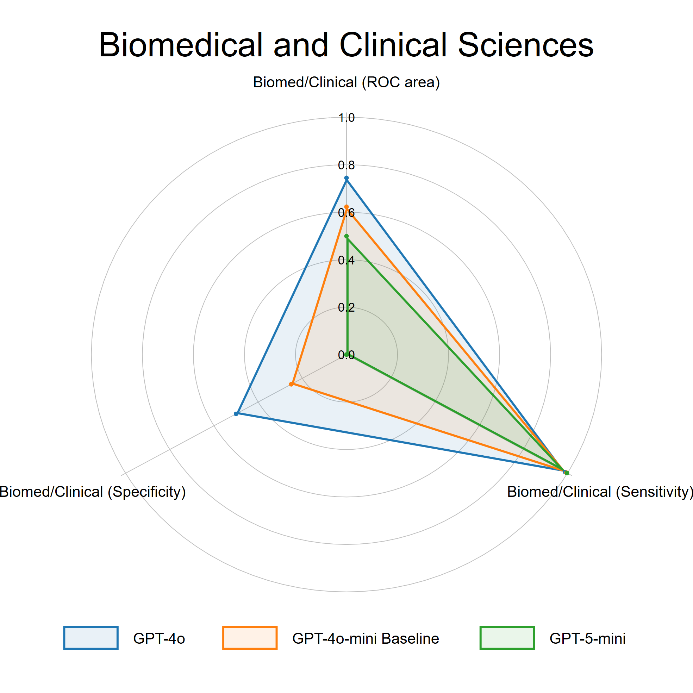


(c) (d)


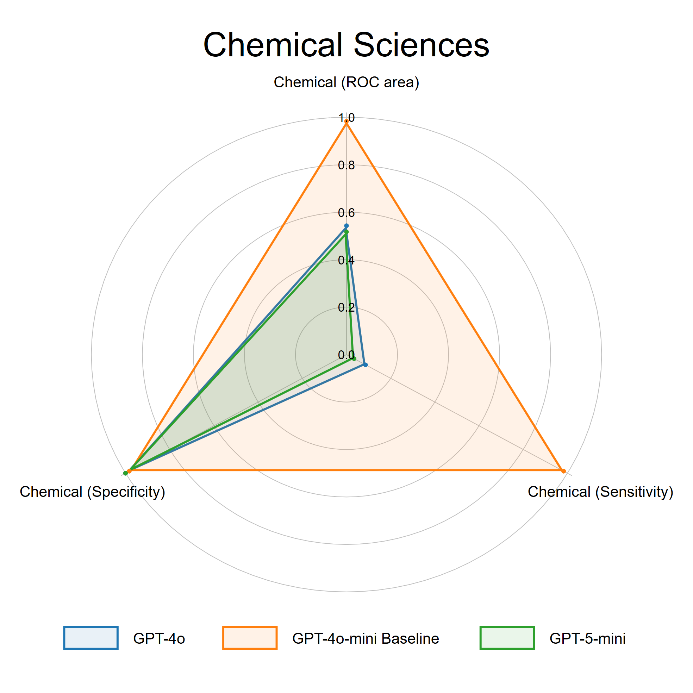

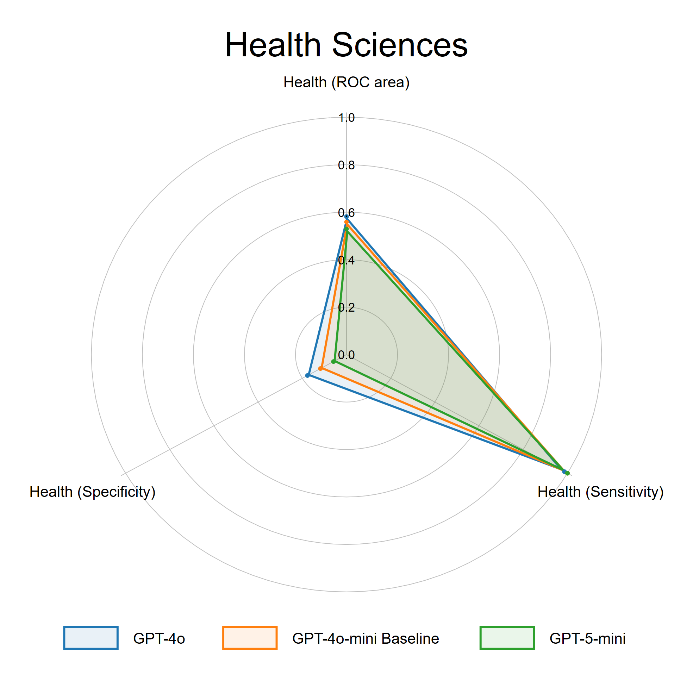


**Fig. S3: Descriptive analysis of the excluded corpus.**


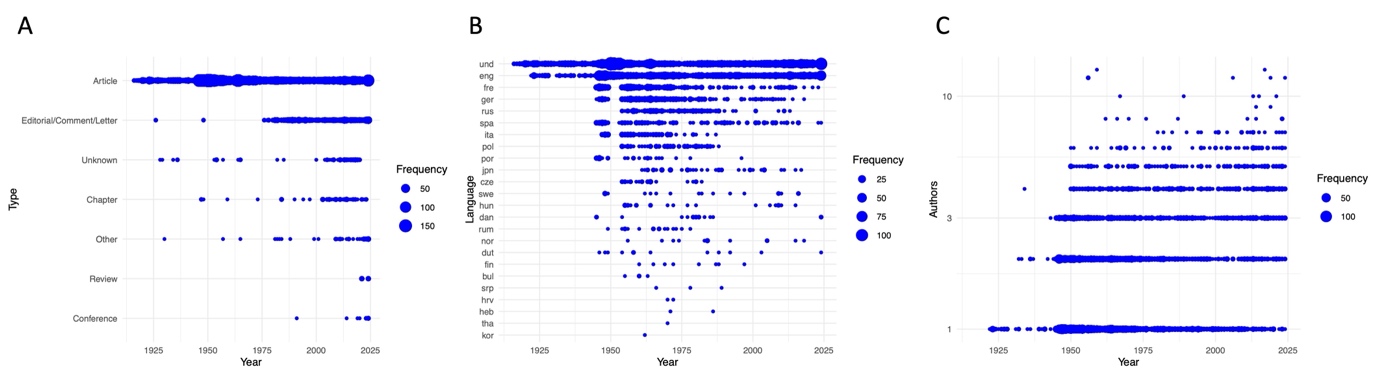


A focus on the distribution of excluded records is made by publication type (A), by language (B), and by coauthor number (C). Bubble sizes represent the relative frequency of occurrence for each category, highlighting trends in record attributes.

**Table S1: Subject heading terms included under the keyword NS in the databases**

| Database | Subject Heading “NS” included terms |
| --- | --- |
| *PMC* | *Syphilis, Central Nervous System (CNS)*  *General Paralysis*  *General Paralysis of the Insane*  *Paralysis, General*  *General Paresis*  *Paretic Neurosyphilis*  *General Paresis of the Insane*  *Juvenile Paresis*  *Neurosyphilis, Asymptomatic*  *Neurosyphilis, Gummatous*  *Neurosyphilis, Juvenile*  *Neurosyphilis, Secondary*  *Secondary Neurosyphilis*  *Neurosyphilis, Symptomatic* |
| *Cochrane* | *Exact Term Match*  *Neurosyphilis*  *Synonyms: Neurosyphilis, Gummatous; Neurosyphilis, Symptomatic; Neurosyphilis, Juvenile; Neurosyphilis, Secondary; Secondary Neurosyphilis; Juvenile Paresis; Neurosyphilis, Asymptomatic; General Paralysis of the Insane; General Paresis of the Insane; Paralysis, General; Paretic Neurosyphilis; General Paralysis; General Paresis; Syphilis, CNS; Syphilis, Central Nervous System; Central Nervous System Syphilis*  *Phrase Matches*  *Neurosyphilis*  *Synonyms: Secondary Neurosyphilis; Neurosyphilis, Secondary; Neurosyphilis, Asymptomatic; Neurosyphilis, Symptomatic; Neurosyphilis, Juvenile; Paretic Neurosyphilis; Neurosyphilis, Gummatous*  *Tabes Dorsalis*  *Synonyms: Tabetic Neurosyphilis; Neurosyphilis, Tabetic*  *Any Word Match*  *Neurosyphilis*  *Synonyms: General Paresis of the Insane; General Paralysis of the Insane; Syphilis, Central Nervous System; Central Nervous System Syphilis; Secondary Neurosyphilis; Neurosyphilis, Secondary; Neurosyphilis, Asymptomatic; Juvenile Paresis; Syphilis, CNS; Neurosyphilis, Symptomatic; Neurosyphilis, Juvenile; Paralysis, General; General Paresis; General Paralysis; Paretic Neurosyphilis; Neurosyphilis, Gummatous*  *Tabes Dorsalis*  *Synonyms: Syphilis, Spinal Meningovascular; Meningovascular Syphilis, Spinal; Spinal Meningovascular Syphilis; Spinal Cord Syphilis; Syphilis, Spinal Cord; Tabetic Neurosyphilis; Neurosyphilis, Tabetic; Spinalis, Tabes; Tabes Spinalis; Locomotor Ataxias; Syphilitic Meningomyelitis; Meningomyelitis, Syphilitic; Ataxias, Locomotor; Syphilitic Meningomyelitides; Ataxia, Locomotor; Meningomyelitides, Syphilitic; Locomotor Ataxia; Myelosyphilis* |
| *Embase* | *'central nervous system lues' OR 'central nervous system syphilis' OR 'CNS lues' OR 'CNS syphilis' OR 'lues cerebro-spinalis' OR 'lues cerebrospinalis' OR 'nervous syphilis' OR 'neuro-lues' OR 'neuro-syphilitic' OR 'neurological lues' OR 'neurological syphilis' OR 'neurolues' OR 'neurosyphilitic' OR 'syphilis, nervous system' OR 'neurosyphilis'* |
| *Web of Science* | *NA* |
| *Lens* | *Identical to PMC* |

The table presents the various synonyms and related terms used for indexing NS-related literature in each database. These variations highlight differences in search terminologies across databases, impacting literature retrieval strategies.

**Table S2. Comparison of classifiers for Task 1 (Topical relevance screening, 10 % validation subset, n = 541) with the baseline of 5411 records classified on 04 Apr 2025, model version ‘gpt-4o mini-2024-07-18’). Analyzed metrics are the agreement between new classifier and the baseline, the total estimated cost and total runtime.**

| Classifier (Model version) | Agreement (%) | Runtime (min) | Estimated Cost (USD) | | Records processed |
| --- | --- | --- | --- | --- | --- |
| GPT-4o mini (‘gpt-4o mini-2024-07-18’ ) | 99.1 | 14  (avg 1.55 s/record) | | 0.04 | 541 |
| GPT-4o (‘gpt-4o-2024-08-06’ ) | 98.3 | 14  (avg 1.55 s/record) | | 1.32 | 541 |
| GPT-5 mini (‘gpt-5 mini-2025-08-07’ ) | 89.4 | 43  (avg 4.77 s/record) | | 0.25 | 541 |
| Human reviewer (Master’s-level assistant) | 96.7 | 135  (avg 14.97 s/record) | | 86.60 | 541 |

**Table S3. Comparison of classifiers for Task 2 (Research field classification, n=471 records identified in Task 1). Analyzed metrics are the average agreement across all 23 FoR, the total estimated cost, and the total runtime.**

| Classifier (Model version) | Average agreement (%) | Runtime (min) | Estimated Cost (USD) | Records×FoR pairs processed |
| --- | --- | --- | --- | --- |
| GPT-4o mini (‘gpt-4o mini-2024-07-18’ ) | 98.2 | 242.72  (avg 1.34 s/pair) | 0.96 | 10833 |
| GPT-4o (‘gpt-4o-2024-08-06’ ) | 94.0 | 261.36  (avg 1.37 s/pair) | 29.60 | 10833 |
| GPT-5 mini (‘gpt-5 mini-2025-08-07’ ) | 97.7 | 716.23  (avg 3.97 s/pair) | 4.66 | 10833 |
| Human reviewer (Master’s-level assistant) | 97.9 | 435  (avg 2.41 s/pair) | 269.40 | 10833 |

**Table S4: Interrater agreement statistics for classification tasks**

| Field Name | Agreement (%) | Expected Agreement (%) | κ | Std· Err· | p value | 95 % CI Lower | 95 % CI Upper |
| --- | --- | --- | --- | --- | --- | --- | --- |
| NS Main Topic | 99·67 | 75·70 | 0·99 | 0·0136 | <0·001 | 99·47 | 99·80 |
| Agricultural, Veterinary and Food Sciences | 99·85 | 98·87 | 0·87 | 0·015 | <0·001 | 99·69 | 99·94 |
| Biological Sciences | 95·42 | 70·78 | 0·84 | 0·015 | <0·001 | 94·77 | 96·00 |
| Biomedical And Clinical Sciences | 99·83 | 98·50 | 0·88 | 0·015 | <0·001 | 99·66 | 99·93 |
| Built Environment and Design | - | - | - | - | - | - | - |
| Chemical Sciences | 98·21 | 80·41 | 0·91 | 0·015 | <0·001 | 97·80 | 98·57 |
| Commerce, Management, Tourism and Services | 100·00 | 1·00 | 1·00 | 0·015 | <0·001 | 99·92 | 100* |
| Creative Arts and Writing | 99·94 | 98·74 | 0·95 | 0·015 | <0·001 | 99·81 | 99·99 |
| Earth Sciences | - | - | - | - | - | - | - |
| Economics | 99·98 | 99·55 | 0·87 | 0·015 | <0·001 | 99·88 | 99·99 |
| Education | 99·98 | 99·55 | 0·95 | 0·015 | <0·001 | 99·81 | 99·99 |
| Engineering | 99·98 | 99·63 | 1·00 | 0·015 | <0·001 | 99·88 | 99·99 |
| Environmental Sciences | 100·00 | 99·83 | 1·00 | 0·015 | <0·001 | 99·60 | 100* |
| Health Sciences | 99·78 | 98·50 | 0·86 | 0·015 | <0·001 | 99·60 | 99·90 |
| History, Heritage and Archaeology | 99·83 | 96·49 | 0·95 | 0·015 | <0·001 | 99·66 | 99·93 |
| Human Society | 98·45 | 81·30 | 0·92 | 0·015 | <0·001 | 98·05 | 98·79 |
| Indigenous Studies | 100·00 | 99·61 | 1·00 | 0·015 | <0·001 | 99·92 | 100* |
| Information And Computing Sciences | 100·00 | 99·61 | 1·00 | 0·015 | <0·001 | 99·92 | 100* |
| Language, Communication and Culture | 99·91 | 99·31 | 0·87 | 0·015 | <0·001 | 99·78 | 99·98 |
| Law And Legal Studies | 100·00 | 99·96 | 1·00 | 0·015 | <0·001 | 99·92 | 100* |
| Mathematical Sciences | 99·94 | 98·70 | 0·95 | 0·015 | <0·001 | 99·81 | 99·99 |
| Philosophy And Religious Studies | 99·98 | 99·63 | 0·94 | 0·015 | <0·001 | 99·88 | 99·99 |
| Physical Sciences | 99·81 | 98·87 | 0·83 | 0·015 | <0·001 | 99·63 | 99·91 |
| Psychology | 99·25 | 84·57 | 0·95 | 0·015 | <0·001 | 98·95 | 99·47 |

The table presents agreement statistics between the two independent classification runs for the task 1 (first row “NS Main Topic”) and for the task 2 (rows 2-24). Studied metrics include the percentage of agreement between classifications, the expected agreement under chance, Cohen’s kappa statistic (which measures agreement beyond chance), its standard error, the p value, and the 95 % CI. The number of classified records is 4 646. (*) One-sided, 97·5 % CI.

**Table S5: Results from the ITSA comparing the impact of clinical discoveries on publication trends in biomedical and clinical sciences versus chemistry**

| Model | Time Period | Coeff | Coeff value | Std Error | p value | 95 % CI Lower | 95 % CI Upper |
| --- | --- | --- | --- | --- | --- | --- | --- |
| Clinical discoveries |  |  |  |  |  |  |  |
|  | Pre 1945 | Initial level | 2·20 | 0·480 | <0·001 | 1·44 | 3·38 |
|  | Pre 1945 | Trend | 1·00 | 0·011 | 0·691 | 0·97 | 1·02 |
|  | Pre 1945 | Field effect | 2·87 | 0·929 | 0·001 | 1·52 | 5·41 |
|  | Pre 1945 | Field × trend | 1·01 | 0·018 | 0·614 | 0·97 | 1·05 |
| *Penicillin G* | 1945 | Change | 2·49 | 0·632 | <0·001 | 1·51 | 4·09 |
|  | Post 1945 | Trend | 0·99 | 0·017 | 0·394 | 0·95 | 1·02 |
|  | 1945 | Field × level change | 0·33 | 0·161 | 0·024 | 0·12 | 0·86 |
|  | Post 1945 | Field × trend change | 1·04 | 0·028 | 0·152 | 0·99 | 1·10 |
| *HIV* | 1981 | Change | 2·37 | 0·911 | 0·025 | 1·11 | 5·03 |
|  | Post 1981 | Trend | 1·00 | 0·023 | 0·990 | 0·96 | 1·05 |
|  | 1981 | Field × level change | 0·77 | 0·371 | 0·591 | 0·30 | 1·98 |
|  | Post 1981 | Field × trend change | 0·99 | 0·029 | 0·824 | 0·94 | 1·05 |
| *Genome* | 1998 | Change | 0·74 | 0·260 | 0·392 | 0·37 | 1·47 |
|  | Post 1998 | Trend | 1·09 | 0·025 | <0·001 | 1·04 | 1·14 |
|  | 1998 | Field × level change | 1·27 | 0·489 | 0·542 | 0·59 | 2·70 |
|  | Post 1998 | Field × trend change | 0·96 | 0·024 | 0·134 | 0·92 | 1·01 |
| Postevent linear trends difference |  |  |  |  |  |  |  |
|  | 1945 | Postevent trend diff· | 0·05 | 0·020 | 0·017 | 0·01 | 0·09 |
|  | 1981 | Postevent trend diff· | 0·04 | 0·022 | 0·055 | 0·00 | 0·08 |
|  | 1998 | Postevent trend diff· | 0·00 | 0·015 | 0·780 | -0·02 | 0·03 |
| Research sharing |  |  |  |  |  |  |  |
|  | Pre 1971 | Initial level | 2·45 | 0·407 | <0·001 | 1·77 | 3·39 |
|  | Pre 1971 | Trend | 1·00 | 0·007 | 0·673 | 0·99 | 1·02 |
|  | Pre 1971 | Field effect | 3·06 | 0·713 | <0·001 | 1·94 | 4·83 |
|  | Pre 1971 | Field × trend | 0·99 | 0·009 | 0·455 | 0·98 | 1·01 |
| *MEDLINE* | 1971 | Change | 1·27 | 0·443 | 0·501 | 0·64 | 2·52 |
|  | Post 1971 | Trend | 1·01 | 0·013 | 0·295 | 0·99 | 1·04 |
|  | 1971 | Field × level change | 2·07 | 0·904 | 0·097 | 0·88 | 4·87 |
|  | Post 1971 | Field × trend change | 1·03 | 0·018 | 0·052 | 1·00 | 1·07 |
| *PMC* | 1999 | Change | 0·76 | 0·261 | 0·419 | 0·38 | 1·49 |
|  | Post 1999 | Trend | 0·96 | 0·048 | 0·454 | 0·87 | 1·06 |
|  | 1999 | Field × level change | 0·76 | 0·305 | 0·497 | 0·35 | 1·67 |
|  | Post 1999 | Field × trend change | 1·11 | 0·060 | 0·053 | 1·00 | 1·24 |
| *Open science* | 2010 | Change | 2·18 | 0·641 | 0·008 | 1·22 | 3·88 |
|  | Post 2010 | Trend | 1·09 | 0·055 | 0·089 | 0·99 | 1·20 |
|  | 2010 | Field × level change | 0·63 | 0·200 | 0·149 | 0·34 | 1·18 |
|  | Post 2010 | Field × trend change | 0·85 | 0·047 | 0·003 | 0·76 | 0·95 |
| Postevent linear trends Difference |  |  |  |  |  |  |  |
|  | 1971 | Postevent trend diff· | 0·03 | 0·014 | 0·060 | 0·00 | 0·05 |
|  | 1999 | Postevent trend diff· | 0·13 | 0·052 | 0·011 | 0·03 | 0·23 |
|  | 2010 | Postevent trend diff· | -0·03 | 0·016 | 0·064 | -0·06 | 0·00 |

The model estimates prevent trends, immediate changes at intervention points, and postevent trends. Key events include the introduction of penicillin G (1945), HIV emergence (1981), and sequencing of the *Treponema pallidum* genome (1998). The table presents coefficients (coeff), standard (std) errors, p values, and 95 % CI. Postevent linear trends and their differences between research fields are also reported. The total number of compared records is N=190.
